# Role of proinflammatory mutations in peri-implantitis: systematic review and meta-analysis

**DOI:** 10.1186/s40729-022-00400-y

**Published:** 2022-01-21

**Authors:** Irene Lafuente-Ibáñez de Mendoza, Amaia Setien-Olarra, Ana María García-De la Fuente, José Manuel Aguirre-Urizar, Xabier Marichalar-Mendia

**Affiliations:** 1 Department of Stomatology II, University of the Basque Country (UPV/EHU), Leioa, Spain; 2 Department of Nursery I, University of the Basque Country (UPV/EHU), Barrio Sarriena s/n, 48940 Leioa, Spain

**Keywords:** Inflammation, Meta-analysis peri-implantitis, Single nucleotide polymorphism, Systematic review

## Abstract

**Purpose:**

To perform a systematic review and meta-analysis on the presence of inflammatory polymorphisms in patients with peri-implantitis (PI). PI is the main complication associated to dental implant therapy. Although its main risk factors are history of periodontitis, poor plaque control and lack of regular maintenance, genetic susceptibility could also be a determinant factor for its appearance. Single nucleotide polymorphisms (SNP) are small mutations of the DNA that alter the osseointegration of implants. Inflammatory proteins participate in both destruction of the extracellular matrix and reabsorption of the alveolar bone.

**Methods:**

A bibliographical research was made in PubMed, Scopus and Web of Science (keywords: “single nucleotide polymorphism”, “polymorphism”, “periimplantitis”, “SNP” and “implant failure”).

**Results:**

There is a statistically significant association of peri-implant bone loss with the homozygotic model of IL-1β (− 511) (OR: 2.255; IC: 1.040–4.889).

**Conclusions:**

Associations between inflammatory polymorphisms and PI must be taken with caution due to the heterogeneous methodological design, sample size and diagnostic criteria of the studies. Thus, more well-designed studies are needed that analyze the relationship between these and more SNP and PI.

## Introduction

The combination of high numbers for partial and total edentulism [1], together with an average life expectancy of over 70 years for the world population [2], shows that treatment with dental implants is a major health advance. However, the biological complications associated with this therapy, preferably peri-implantitis (PI), are frequent and must be prevented [3].

Peri-implantitis is an infectious and inflammatory multifactorial disease affecting more than 45% of patients with dental implants, which is characterized by progressive loss of the alveolar bone [3]. History of periodontitis, poor plaque control and lack of regular maintenance are the main risk factors of this disorder [4]. Nevertheless, not all individuals with these features end up developing PI. Thus, genetic susceptibility has also been suggested as an important factor in the development of PI [5].

Genetic polymorphisms are individual variations at a given location in the DNA sequence, of which single nucleotide polymorphisms (SNP) are the most common [6]. The detection of SNP can be used to identify altered genes or proteins in a specific disease. Several genotypes of inflammatory proteins are strongly associated with chronic or aggressive periodontitis [7, 8]. In the case of PI, different SNP involved in the inflammatory response have also been studied, mainly IL-1β and IL-1α [9].

IL-1 is a low molecular weight protein that promotes alveolar bone resorption, extracellular matrix destruction and osteoclastogenesis; thus, it plays an important role in bone physiopathology [9]. The most frequent and studied SNP are located at positions 3953 [*IL-1 (*+ *3953)*], 511 [*IL-1 (*− *511)*] [10, 11] and 889 [*IL-1α (*− *889)*]. SNP of other inflammatory molecules include *IL-10 (*− *1081)* y *IL-6 (*− *174)* and *TNF-α (*− *308)* [12–14]. The link between these SNP and peri-implantitis has been highly variable, probably due to differences in the diagnostic criteria. The mutation of these genes could trigger an abnormal inflammatory and resorptive response that decreases the osseointegration of dental implants. Discovering the existence of a specific genotypic profile of certain SNP in patients with peri-implantitis would help us assess the level of individual risk and establish appropriate preventive measures.

With this background, we planned to carry out a systematic review and meta-analysis, with the aim of understanding the relationship between the presence of proinflammatory polymorphisms and the development of peri-implantitis.

## Methods

### Information sources and search strategy

The design of this study matches the PRISMA criteria [15]. Systematic bibliographical research was performed in PubMed (US National Gallery of Medicine), Web of Science/Knowledge and Scopus, with the keywords “single nucleotide polymorphism”, SNP, “peri implantitis”, and “implant failure”: (“single nucleotide polymorphism” AND “peri implantitis”; “single nucleotide polymorphism” AND “implant failure”; SNP AND “peri implantitis”: SNP AND peri-implantitis; SNP AND “implant failure”). A manual search of the referenced studies, as well as of prominent journals of the field, was carried out aiming to include additional papers.

PECOS question was: patients with dental implants (population), with SNP of proinflammatory proteins (exposure), in contrast to patients with dental implants who do not have proinflammatory SNP (comparison), to study the effect of these SNP in the onset of peri-implantitis (outcome). Only longitudinal observational studies were included (type of study).

### Eligibility criteria

The articles selected for this work met the following inclusion criteria: (1) being published until October 2021; (2) being written in English or Spanish (guarantee of full comprehension of content); (3) human studies. Exclusion criteria were: (1) studies that did not analyze proinflammatory polymorphisms and/or did not show the genotype frequencies; (2) studies on peri-implant disease that did not report the peri-implant bones loss; (3) previous meta-analysis or reviews, and (4) case reports, conferences or chapter of books. The information extracted from each study was: author and year of publication, type of study, number of patients (with and without PI) and genotype frequency of the polymorphisms.

### Selection process

Two independent reviewers made a duplicate bibliographical research (ILIM, ASO). Title and abstract of all registers were evaluated, and then, these were analyzed taking into account the inclusion and exclusion criteria. Any disagreement between the them was resolved by a third reviewer (XMM) to minimize risk of bias. Data about the included studies were gathered by two reviewers (ILIM, ASO) and double-checked by another three (XMM, AMGF, JMAU), to guarantee the integrity of the contents.

### Quality analysis

We used modified Newcastle-Ottawa Scale (NOS) [16] to assess the methodological quality of the included studies. This system analyzes the risk of bias of nonrandomized studies, taking into account three domains and eight items for case–control studies: selection, comparability and outcome. The total maximum score is 10; a study with a score from 8 to 10 has high quality; 4 to 7, high risk of bias; and 0 to 3, very high risk of bias.

### Statistical analysis

To analyze the heterogeneity of the studies, *I*^2^ test was applied. Fixed-effect model was used when *I*^2^ < 50%. To evaluate the correlation of PI with the susceptibility to different genotypes, the following genotypic models were carried out: heterozygous model (T/C vs T/T) and homozygous model (C/C vs T/T). For each model the odds ratio (OR) and 95% confidence interval (CI 95%) were obtained. Statistical analysis was performed with the OpenMeta tool (Analyst).

## Results

### Results of the search

We obtained a total of 192 records in the initial research, out of which 103 were eliminated because they were duplicates. Additionally, three articles were included by manual search. After the initial screening, 47 articles were excluded: 43 for not investigating the presence of SNP in PI and 4 for not being available in full-text. Thus, 45 registers were analyzed for their suitability, but 15 were eliminated: 13 because they were previous meta-analyses or literature reviews and another 2 because they were conference texts or book chapters. Also, we excluded 13 registers that did not analyze inflammatory polymorphisms and 5 that did not indicate the parameters used for the diagnosis of peri-implant disease. Finally, 12 studies were selected for the systematic review, whose data are shown in Table 1 [10–14, 17–23].

**Table 1:** Main data of the included studies

Authors, year	Patients			Diagnostic criteria for diagnosis of peri-implantitis
	Ethnicity	Case	Control	
Shimpuku et al. 2003	Japan	17	22	ABL > 0.5 mm
Laine et al. 2006	Belgium	71	44	ABL (3 threads), BOP, pus
Cury et al. 2007	Brazil	17	19	ABL (3 threads), BOP, pus
Lachmann et al. 2007	Germany	11	18	PPD > 4 mm
Lin et al. 2007	Japan	29	30	ABL > 0,5 mm
Hamdy et al. 2007	Egipt	25	25	PPD > 4 mm, ABL, BOP
Gurol et al. 2011	Turkey	32	46	ABL > 3 mm, PPD > 5 mm, BOP, pus
Melo et al. 2012	Italy	16	31	PPD > 4 mm, BOP, pus
Ladeira-Casado et al. 2013	Brazil	31	40	ABL > 3 mm, pus
García-Delaney et al. 2015	Spain	27	27	ABL > 2 mm, PD > 4 mm, BOP, pus
Rakic et al. 2015	Germany	189	180	ABL (> 2 threads), PPD > 5 mm, BOP
Petkovic-Curcin et al. 2017	Serbia	34	34	ABL (> 2 threads), PPD > 4 mm, BOP

Case: patients with peri-implantitis. Control: patients without peri-implantitis. ABL: alveolar bone loss; BOP: bleeding on probing; PPD: peri-implant pocket depth

**Fig. 1:**
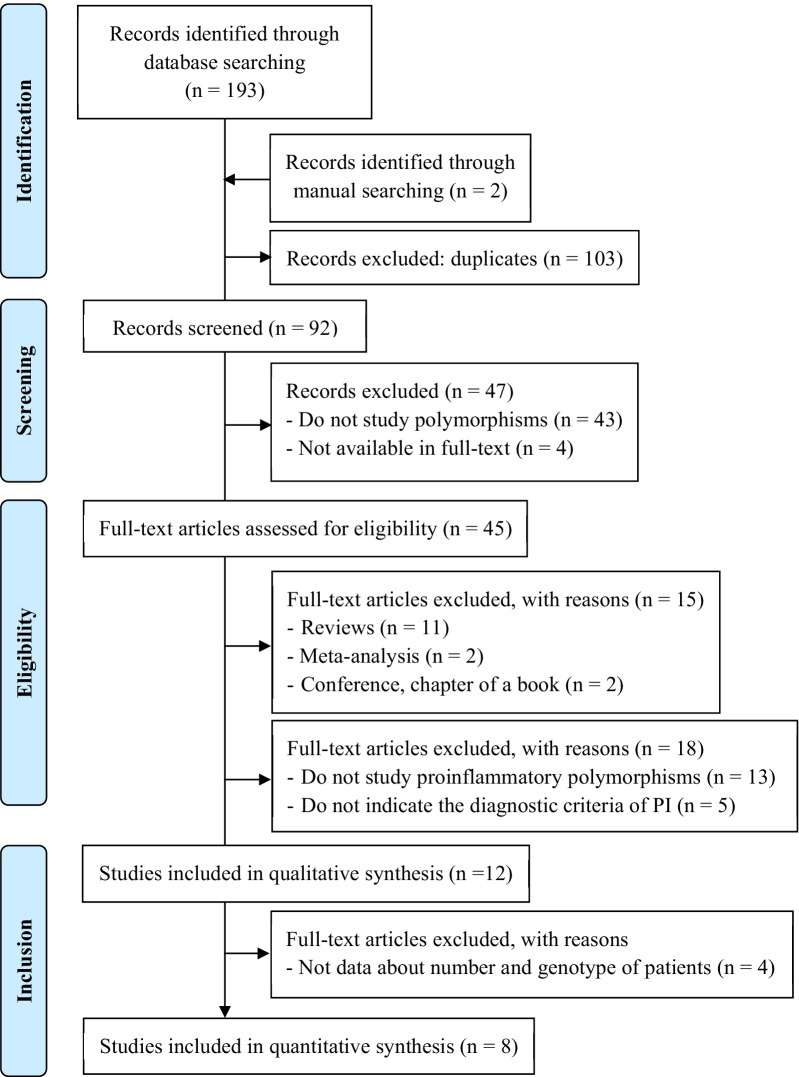
PRISMA flow diagram. Synthesis of the selection process of the included articles in the review

We were only able to use eight studies to perform the meta-analysis, due to lack of genotype data [10, 12, 13, 17–19, 21–23]. The summary of the selection process is shown in Fig. 1.

### Characteristics of included studies

In total, the included studies investigated 1015 patients, of 499 with peri-implantitis 516 without peri-implantitis. From these, 98 were from Asia (China and Japan) and the rest from other countries of Europe (Austria, Belgium, Germany, Italy, Turkey, Spain), America (Brazil) and Africa (Egypt). The authors used either radiographic alveolar bone loss (ABL) [10–14, 17, 19, 20, 22, 23] or peri-implant pocket depth (PPD) analysis [18, 21] to make the diagnosis (Table 1).

#### IL-1β (+ 3953) and IL-1β (− 511)

Most of the studies included in this review analyze the possible relationship between the presence of SNP IL-1β (+ 3953) and the development of PI [10, 11, 17–21]. However, only two studies [10, 20] observed a statistically significant association between the composite genotype IL-1β (+ 3945) and IL-1α (− 889), and patients with PI.

The link of SNP IL-1β (− 511) and peri-implantitis has been analyzed in four studies [10, 17, 19, 20], but only two conducted in Japan [17, 19] recognized a direct relation to peri-implant bone loss.

#### IL-10 (− 1081) y IL-6 (− 174)

The IL-10 SNP (− 1081) was assessed in two studies [13, 23], and it was only associated to PI in German smoker patients or with a history of periodontitis [23]. On the contrary, IL-6 SNP (− 174) was only associated with PI in Serbian individuals [14, 21, 23].

#### TNF-α (− 308)

TNF-α genotype (− 308) was only linked to peri-implant disease in Serbian patients [22, 23], and not in Brazilians [12, 13].

### Meta-analysis

**Fig. 2:**
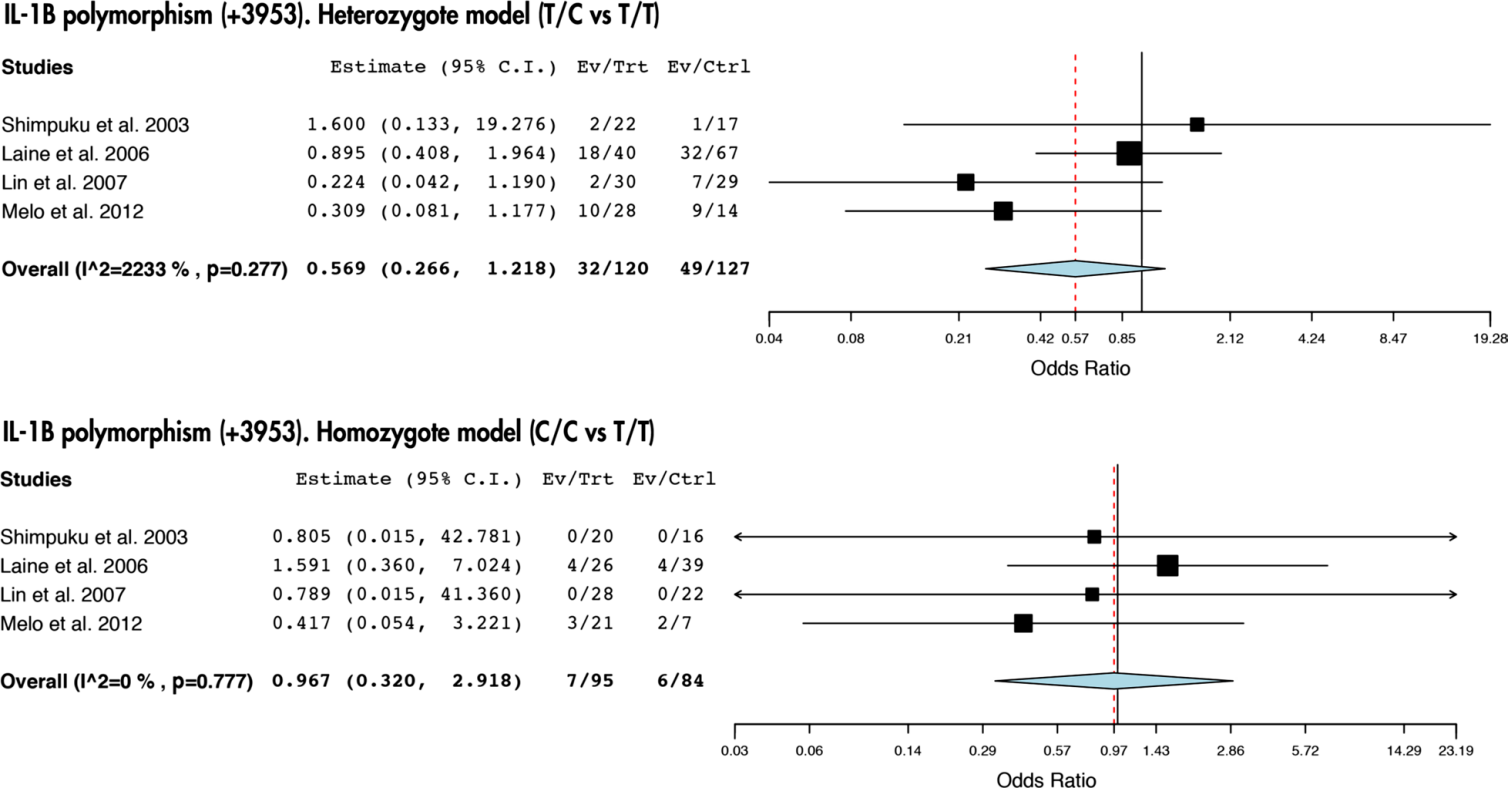
Association between IL-1β (+ 3953) polymorphism and peri-implantitis in the heterozygote and homozygote model

**Fig. 3:**
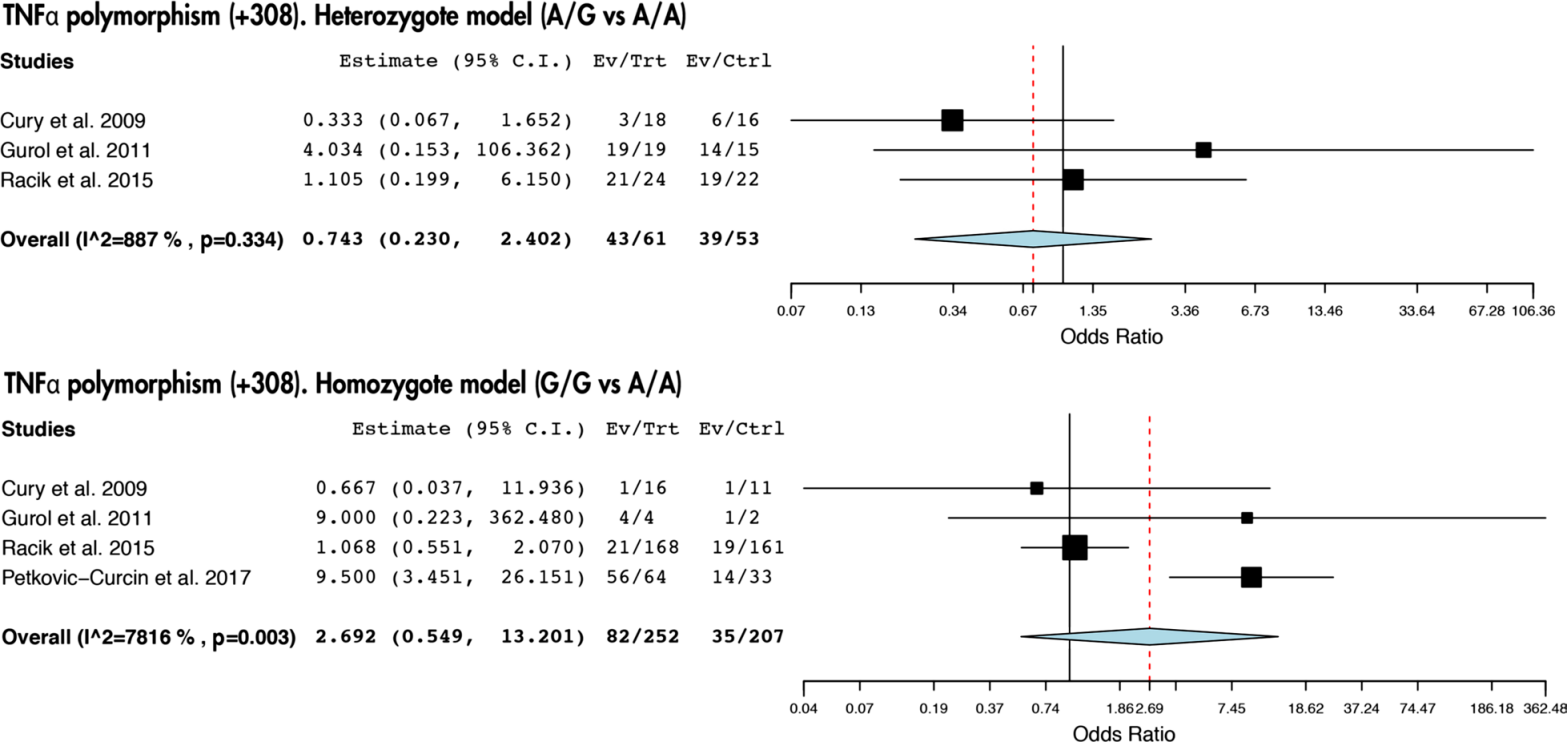
Association between TNFα (− 308) polymorphism and peri-implantitis in the heterozygote and homozygote model

**Fig. 4:**
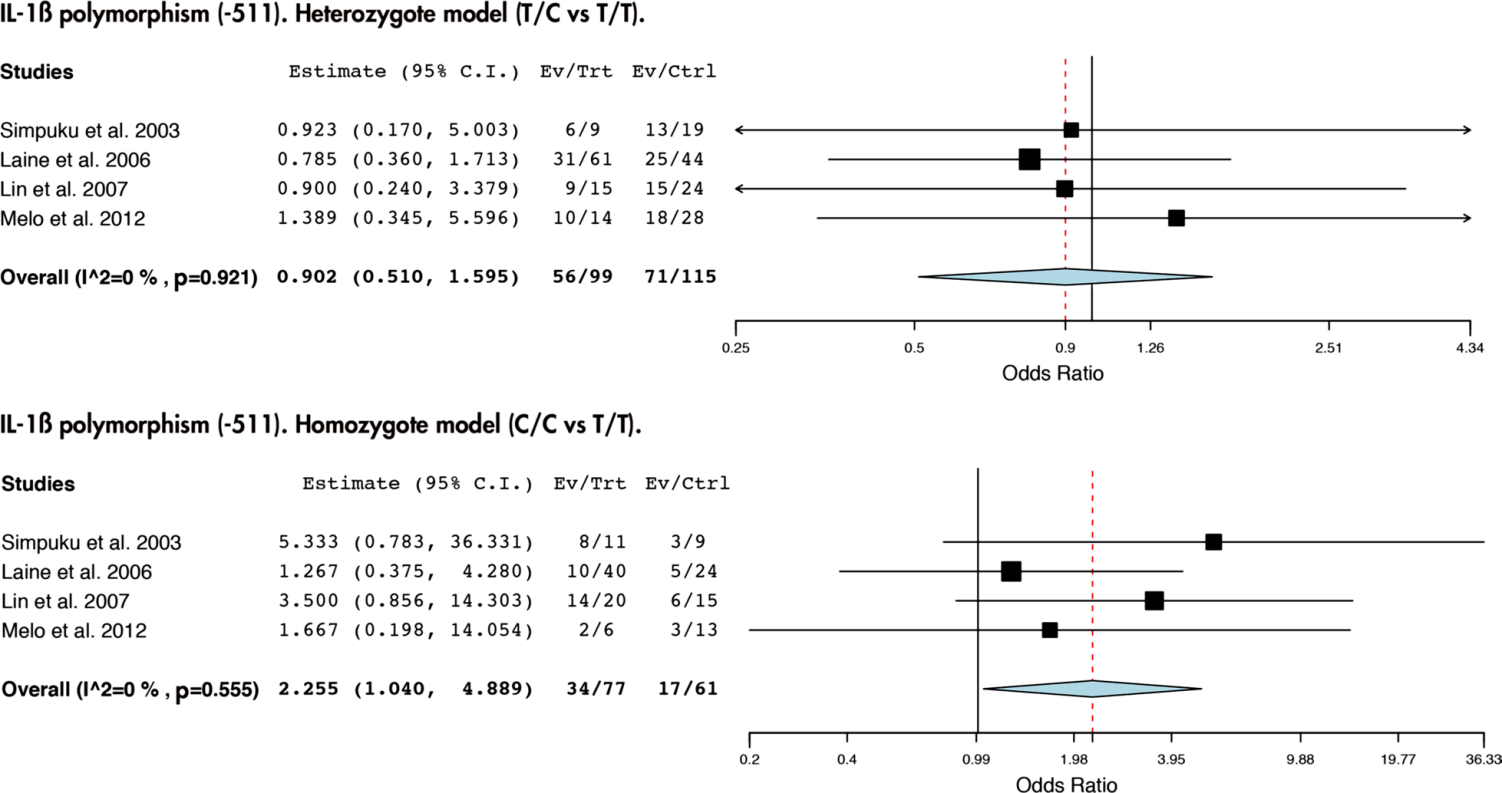
Association between IL-1β (− 511) polymorphism and peri-implantitis in the heterozygote and homozygote model

Our study revealed that there are no statistically significant link between the presence of IL-1β (+ 3953) nor TNF-α (− 308) polymorphisms and peri-implantitis (Figs. 2 and 3). However, we did observe a risk association between the presence of the C/C genotype of the SNP IL-1β (− 511) and peri-implantitis (T/C vs T/T: *I*^2^ = 0%, *p* = 0.921; OR: 0.902; IC 95% 0.510–1.595 and C/C vs T/T: *I*^2^ = 0%, *p* = 0.555; OR: 2.255; IC 95%: 1.040–4.889) (Fig. 4).

### Quality of studies

After applying modified NOS assessment, 16.67% of the studies revealed 8 stars and 83.3% of them 6 (Table 2). Overall risk of bias was low.

**Table 2:** Quality assessment of the included studies: NOS tool

Authors, year	Type of study	New Castle-Ottawa Scale (NOS)		
		Selection	Comparability	Exposure
Shimpuku et al. 2003	Case–control	★★★	★	★★
Laine et al. 2006	Case–control	★★★	★★	★★
Cury et al. 2007	Case–control	★★★	★	★★
Lachmann et al. 2007	Case–control	★★★	★	★★
Lin et al. 2007	Case–control	★★★	★	★★
Hamdy et al. 2007	Case–control	★★★	★	★★
Gurol et al. 2011	Case–control	★★★	★	★★
Melo et al. 2012	Case–control	★★★	★	★★
Ladeira-Casado et al. 2013	Case–control	★★★	★	★★
García-Delaney et al. 2015	Case–control	★★★	★	★★
Rakic et al. 2015	Case–control	★★★	★★	★★
Petkovic-Curcin et al. 2017	Case–control	★★★	★	★★

## Discussion

The first cases of peri-implantitis were described as “inflammatory reactions with loss of supporting bone in the tissues surrounding a functioning implant” [24]. PI is physiopathologically different to periodontitis and has recently been considered as an inflammatory disorder (“Peri-implant Conditions and Diseases”) [25, 26].

Inflammation is a physiological response that participates in many acute and chronic diseases in humans [27]. The term interleukin-1 was firstly used in the International Lymphokine Workshop in Ermatingen in 1979 [28], to define “a macrophagic product that stimulates T and B cells, with non-immunological properties”. Because there is strong evidence of the role of IL-1β in the physiopathology of periodontitis [8], recent research has tried to discover its link to peri-implantitis.

After conducting this review, we found that only two authors demonstrated a significant association between the composite genotype of IL-1β (+ 3945) and IL-1α (− 889) and the presence of peri-implantitis [10, 18]. This genotype has already been associated to patients with chronic periodontitis, but not with aggressive periodontitis [8]. Thus, we believe that there may be only a specific group of patients with peri-implantitis who present this genotypic profile. Myeloid differentiation factor-88 (MyD88) is responsible for the activation of proinflammatory cytokines IL1β and IL-1α, inducing at the same that an intracellular cascade system that secretes both proteins to the extracellular matrix [29]. Unlike IL-1β, IL-1α has a silent nuclear expression under normal homeostasis that changes during the inflammatory response [29]. This may explain why IL-1α polymorphism [IL-1α (− 899)] alone is not independently associated with the development of PI.

During the eligibility analysis, several studies were excluded because they did not indicate the diagnostic criteria of PI. From these, Feloutzis et al. [30] and Gruica et al. [31] found a significant association between SNP IL-1β (+ 3953) and peri-implant bone loss in heavy smokers (> 20 cigarettes/day). However, they did not indicate whether the patients had peri-implantitis. Furthermore, our analysis did not find a significant association between IL-1β (+ 3953) polymorphism, tobacco use and PI [15, 16, 25, 27].

In contrast, two study groups from Japan reported a strong link between IL-1β (− 511) and PI [17, 19], which remained significant at the final meta-analysis (Fig. 4). This positive result could respond to the fact that some authors used ABL > 0.5 mm as diagnostic criteria for PI (Table 1). Although the first sign of peri-implantitis can be the presence of a bone loss (0.5 mm) [32], diagnosis of PI is based on: (1) presence of bleeding and/or suppuration on gentle probing, (2) probing depths of ≥ 6 mm, or bigger than previous examinations, and (3) bone levels ≥ 3 mm apical of the most coronal portion of the intraosseous part of the implant, or greater than initial bone remodeling [26]. Therefore, the PI cases of several studies [17, 19] could currently be reclassified as peri-implant health and more studies are needed to further clarify this relationship. Furthermore, the fact that the other studies that also analyzed this SNP did not observe such link, with more precise diagnostic criteria of PI (Table 1), make us believe that the C/C genotype of IL-1β (− 511) would truly be associated to alveolar bone loss and not actual peri-implantitis [10, 21].

The diagnostic criteria of peri-implantitis have been in constant change throughout the years, and it is not possible to ensure that all the patients were correctly classified as either healthy or sick. Since these variations are very important in risk assessment studies, the application of the latest classification of periodontal diseases may reduce this bias, allowing the homogeneity of future investigations [33].

IL-10 is a potent anti-inflammatory cytokine that reduces the synthesis of proinflammatory chemokines (IL-1, TNF-α) and extracellular matrix proteins (gelatinase, collagenase), while enhancing osteoblast differentiation and inhibiting osteoclast formation [34]. Mutations in its gene could affect bone homeostasis. However, the IL-10 SNP (− 1081) has only been associated with patients who smoke or have a history of periodontitis [23]. In contrast, IL-6 has a dual role in bone remodeling. Under normal conditions, it suppresses bone resorption by inhibiting the differentiation of osteoclast progenitors, and under inflammatory conditions it induces RANKL expression in osteoblasts and facilitates the proliferation of osteoclast progenitors [35]. Thus, alterations on IL-6 gene need to be evaluated together with those of other inflammatory markers. This may explain why the IL-6 SNP (− 174) does not affect all individuals equally [23].

A proinflammatory cytokine that also plays an essential role in bone remodeling and homeostasis is tumor necrosis factor-α (TNF-α), suppressing osteoblastic proliferation and activating osteoclastogenesis from its early stage, when marrow-derived macrophages are still osteoclast precursor cells [36]. In this work only few authors have recognized a relation between the TNF-α (− 308) SNP and peri-implantitis [22, 23]. Since the meta-analysis confirmed this association as significant, further studies with more patients will be needed.

This systematic review has some limitations. First, the evidence level of the included studies was low (class III) [37]. Therefore, the reliability of our conclusions might be low. And second, the sample sizes in the investigated studies were small. High-evidence SNP studies normally need very more patients and, thus, the power analysis might be as low as 5%. Taking all this into account, it is necessary to plan more well-designed studies with larger samples, in order to further analyze the involvement of these genetic polymorphisms and more inflammatory molecules involved in peri-implant processes.

## Conclusions

In summary, after performing this systematic review and meta-analysis we conclude that, currently, there is no evidence that patients carrying the IL-1β (+ 3945), IL-10 (− 1081), IL-6 (− 174) or TNF-α (− 308) SNPs have a higher risk of developing peri-implantitis. However, individuals with the C/C genotype of the SNP IL-1β (− 511) and those with composite genotype IL1β (+ 3945) and IL-1α (− 889) may have a higher risk for peri-implantitis. Also, patients who smoke more than 20 cigarettes a day and have IL-1β (+ 3953) polymorphism would have a higher risk of peri-implant bone loss.

## Data Availability

All data generated or analyzed during this study are included in this published article [and its additional information files].
